# Street vending and informal economy: Survey data from Cali, Colombia

**DOI:** 10.1016/j.dib.2017.06.047

**Published:** 2017-07-01

**Authors:** Lina M. Martinez, Daniela Estrada

**Affiliations:** Universidad Icesi, Cali, Colombia

**Keywords:** Data, Informal economy, Street vendors, Colombia

## Abstract

This data brief describes data collected in Cali, Colombia about the economic dynamic and socioeconomic conditions of street vendors in the city. The study was conducted between 2014 and 2016 in two populated sites in terms of formal and informal commerce in the city. We present the methodology followed in the study, location of street vending sites and type of data collected to approximate to the economic dimension of street vending. Data collected contains information about sociodemographic characteristics, life satisfaction, business operation and characteristics, income and expenses, official license for operation. This information is linked to the publication (Martinez et al., 2017) [1].

**Specifications Table**

**Table:** 

Subject area	Public Policy
More specific subject area	*Informal Economy*
Type of data	Text, dummy and metric variables
How data was acquired	Survey and observation
Data format	Raw
Experimental factors	None
Experimental features	None
Data source location	Cali - Colombia
Data accessibility	Observatorio de Políticas Públicas – POLIS www.icesi.edu.co/polis/
Related research article	Martínez et al., 2017

**Value of the data**

• This data allows an approach to the economic dynamic of street vending and the conditions of those involved in this informal activity. Information provided in this study is relevant for policy formulation since the regulation of street vending is central to urban planning.
• Data collected in this study can be compared with similar studies conducted about the socioeconomic conditions of street vendors in developing countries. Questions about poverty, education, family composition and expectations are similar to different international studies.
• The data also allows to assess the economic impact of street vending in cities. It is possible to stablish links between formal and informal economies and the flow of money between sectors.
• It is also possible to understand the links between workers in the informal sector and the government. Issues such as tax evasions, access to banking, and participation on welfare programs are tacked.

## 1 Data

The data presented was collected by observational and direct surveys (face-to-face) to street vendors in Cali, Colombia in two street vending sites. First study was conducted during December 2014 in the city downtown. Second study was conducted in 2016 in Santa Helena Market. In both studies street vendors answered questions concerning socioeconomic status, family composition, income (including sales and profits), education, life satisfaction, and access to government welfare. The survey was randomly collected, anonymous, and voluntary.

## 2 Experimental design, materials and methods

**Fig. 1:**
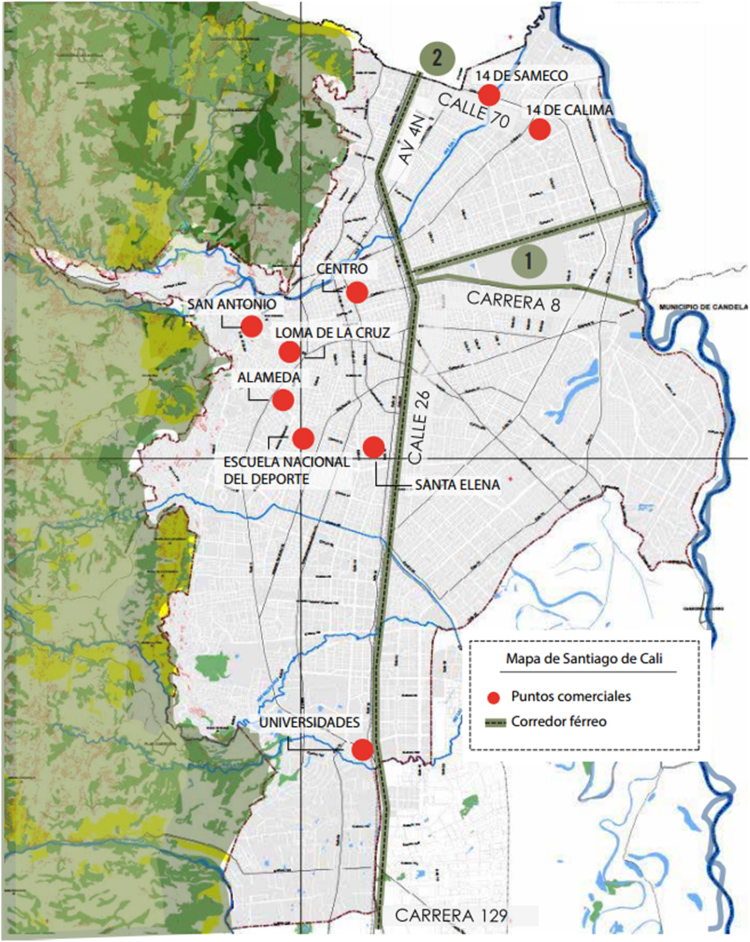
Main locations of street vendors in Cali, Colombia.

According to the local government, there are nine sites in the city where street vendors conduct their economic activity (see Fig. 1 – marked in red). The study took place in two sites: Centro and Santa Helena, the most populated areas in the city in terms of street vendors and formal commerce. Street vending in downtown (centro) covers an important array of cheap merchandise from footwear to cell phone accessories. Santa Helena is a food market.

**Fig. 2:**
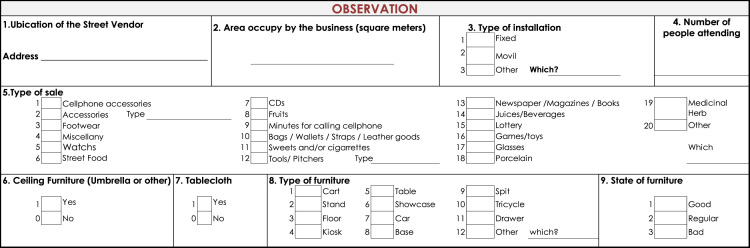
Standardized observational format. Street vendors in Cali, Colombia.

**Fig. 3:**
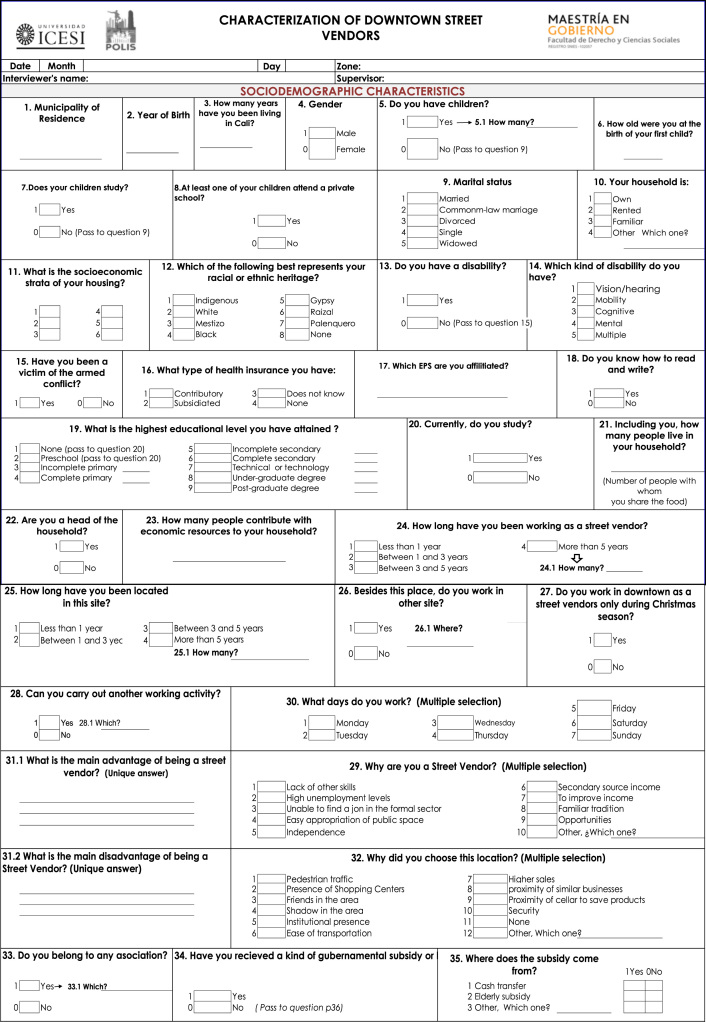

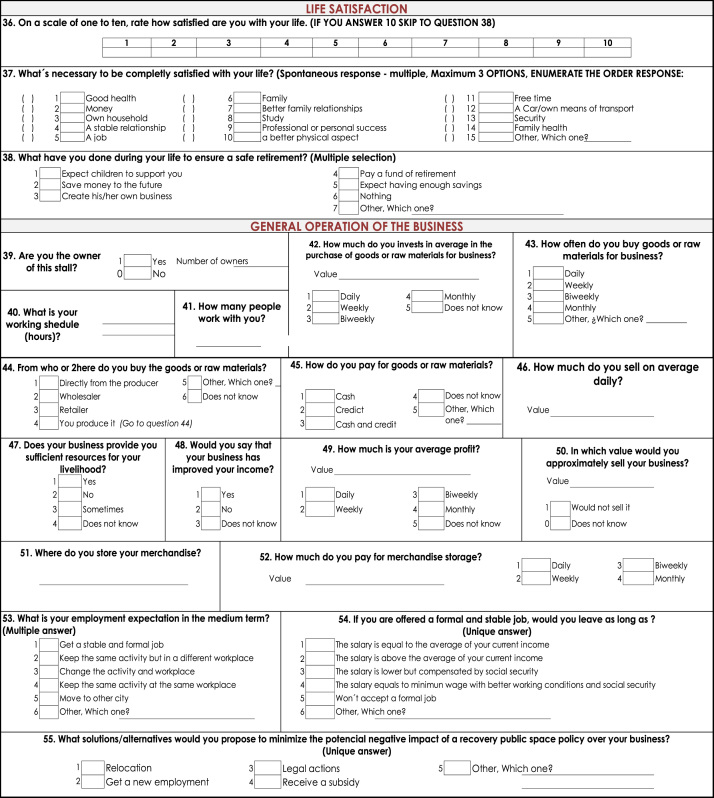

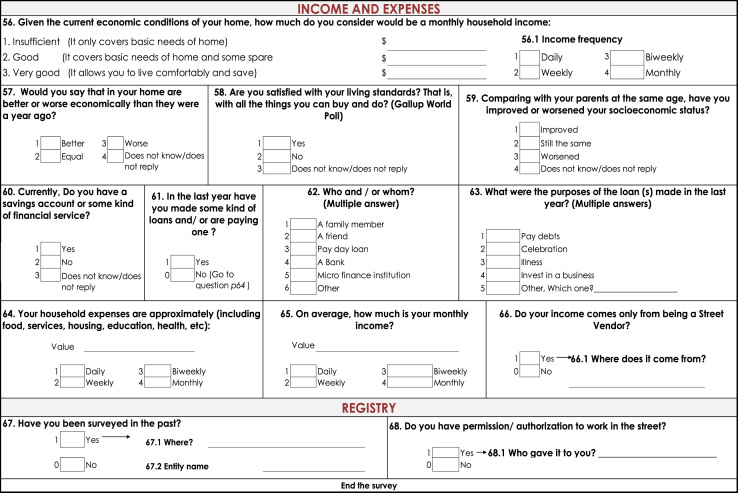
Survey information. Street vendors in Cali, Colombia.

Two types of data were collected: observational and survey data (Figs 2 and 3 present the details). The former was collected using a standardized observational format which collected information on: types of stalls (fixed or mobile), type of products offered, number of people working at a stall, and an indicator of the physical condition of the stall (physical condition was defined as good, fair and poor and its classification was based on the condition of each stall as perceived by the observer). This information allowed to count total number of street vendors and proxy to the occupation in public space. Second type of data was survey information. Respondents answered questions concerning socioeconomic status, family composition, income (including sales and profits), education, life satisfaction and access to government welfare. This information is used to construct variables about business operation and profitability and socioeconomic conditions of street vendors Analysis using this information has been published (Martinez et al., 2017).

This study follows local and international rules for empirical research and is approved by the Institutional Review Board of Universidad Icesi. Likewise, respondents provide verbal consent before survey commencement. Information of this study (Observarorio de Políticas Públicas - POLIS, 2016), available at: www.icesi.edu.co/polis/. There is a policy brief displaying principal findings of this study, available in Spanish in the web page (Fig. 2, Fig. 3).
