# Supplementary material for: Street vending and informal economy: Survey data from Cali, Colombia
Source: Data Brief. 2017 Jul 1;14:132–7. doi: 10.1016/j.dib.2017.06.047 (PMC5537378; doi:10.1016/j.dib.2017.06.047)
Supplement: Supplementary file 1 — Supplementary material [file mmc1.pdf]

Conflicts of Interest Statement

Manuscript title: **Street Vending and Informal Economy: Survey Data from Cali, Colombia**

The authors whose names are listed immediately below certify that they have NO affiliations with or involvement in any organization or entity with any financial interest (such as honoraria; educational grants; participation in speakers' bureaus; membership, employment, consultancies, stock ownership, or other equity interest; and expert testimony or patent-licensing arrangements), or non-financial interest (such as personal or professional relationships, affiliations, knowledge or beliefs) in the subject matter or materials discussed in this manuscript.

Author names:

Lina Martínez

Daniela Estrada

This statement is signed by all the authors to indicate agreement that the above information is true and correct

Name	Signature	Date
Daniela Estrada	Daniela Estrada	26-04-2017
Lina Martinez		26-04-2017
